# 528 Efficacy of Autologous Skin Cell Suspension in the Closure of Thermal and Non-thermal Full-thickness Wounds

**DOI:** 10.1093/jbcr/irae036.163

**Published:** 2024-04-17

**Authors:** Samantha Brown, C Scott Hultman, Carrie McGroarty, Michelle McMoon, Corianne Rogers

**Affiliations:** WakeMed Health and Hospitals, Raleigh, NC; WakeMed Health and Hospitals, Raleigh, NC; WakeMed Health and Hospitals, Raleigh, NC; WakeMed Health and Hospitals, Raleigh, NC; WakeMed Health and Hospitals, Raleigh, NC

## Abstract

**Introduction:**

Autologous Skin Cell Suspension (ASCS) has documented success in the closure of partial-thickness burns, but far less is known about the safety and efficacy of ASCS in the closure of full-thickness injuries. The purpose of this study is to examine the utility of ASCS, when used in combination with widely-meshed skin grafts, in achieving closure of both thermal and non-thermal full-thickness defects and minimizing donor site morbidity.

**Methods:**

In this case series, 40 consecutive patients with full-thickness defects (burns 7, MVC 12, infection 13, other 8) underwent debridement and were treated with the bilayer technique of 3:1 widely-meshed autograft and 80:1 expanded ASCS, from September 2022 through September 2023, at an urban Level 1 trauma center. Single cell suspension of keratinocytes, melanocytes, and fibroblasts underwent point-of-care preparation in the operating theater using trypsin, buffer, and micro-filtration. Aerosolized cells were sprayed over widely-meshed grafts and donor sites, fixated with tissue glue, covered with a non-adherent clear polymer dressing. End points were >99% re-epithelialization by 4, 8, and 12 weeks, rate of limb salvage, size of donor site reduction, operative throughput, time from grafting until discharge and total length of stay, and incidence of early and late postoperative complications.

**Results:**

Percentage of patients with >99% wound closure was 75%, 95%, and 97.5% at 4, 8, and 12 weeks, respectively. Limb salvage was accomplished in 35 out of 36 patients (6 upper, 30 lower extremities). Mean area grafted was 506 cm2, with a donor site of 248 cm2 (reduction of 49%). Mean surgical time (start-to-finish) was 73 minutes, compared to OR time (in-to-out) of 129 minutes. Mean length-of-stay was 23.1 days, while time from ASCS to discharge was 9.5 days. Three out of 40 patients (7.5%) required reoperation for bleeding (1) and breakdown (2), and 4 out of 40 patients (10%) developed hypertrophic scarring. Mean follow-up was 101.5 days.

**Conclusions:**

When used for closure of full-thickness, thermal and non-thermal defects, point-of-care ASCS is effective and safe. Particular benefits include rapid wound re-epithelialization, high rate of limb salvage, reduction of donor site size and morbidity, and low incidence of hypertrophic scarring.

**Applicability of Research to Practice:**

This project demonstrates the efficacy and safety of spray-on skin for closure of full thickness defects.

